# Time for new thinking about sensitive periods

**DOI:** 10.3389/fnsys.2014.00055

**Published:** 2014-04-09

**Authors:** Virginia Penhune, Etienne de Villers-Sidani

**Affiliations:** ^1^Laboratory for Motor Learning and Neural Plasticity, Psychology, Concordia UniversityMontreal, QC, Canada; ^2^International Laboratory for Brain, Music and SoundMontreal, QC, Canada; ^3^Neurology and Neurosurgery, Montreal Neurological InstituteMontreal, QC, Canada

**Keywords:** brain plasticity, early experience, cognitive development, neuro-development, brain maturation

The impact of training or experience is not the same at all points in development. Children who receive music lessons, or learn a second language before age 7–8 are more proficient as adults. Early exposure to drugs or trauma makes people more likely to become addicted or depressed later life. Rat pups exposed to specific frequencies from 9 to 13 days post-partum show expanded cortical representations of these frequencies. Young birds must hear and copy their native song within 1–2 months of birth or they may never learn it at all. These are examples of sensitive periods: developmental windows where maturation and specific experience interact to produce differential long-term effects on the brain and behavior.

While still controversial, evidence for the existence of sensitive periods has grown, as has our understanding of the underlying mechanisms of brain plasticity. Behavioral evidence from studies of language, psychopathology or vision in humans has been complemented by evidence elucidating molecular, gene, and hormonal mechanisms in animals. It has been proposed that sensitive periods can be both opened and closed by specific experience, and that there are multiple, overlapping sensitive periods that occur through-out development as functions come on line. It is also likely that experience-dependent behavioral or brain plasticity accrued during one sensitive period can serve as a scaffold on which later experience and plasticity can build.

Research into sensitive periods—or the interaction between development and specific experience—has entered a new phase as evidenced by the range of contributions brought together in this volume. Until very recently, sensitive periods were considered to be relatively narrow phenomena, often associated with the acquisition of specific perceptual abilities. This narrow definition has now evolved into a broader concept suggesting that the timing of individual experience interacts with developmental changes in the brain to produce synergistic effects on perceptual, cognitive, and motor function.

The broad concept that the timing of individual experience interacts with brain development and might even guide it is illustrated by articles examining both lower and higher-level brain functions, such as the effect of age of start of music training on brain stem responses to speech sounds (Skoe and Kraus, 2013); the effect of age of language acquisition on discrimination of visual speech cues (Weikum et al., 2013) or novel language learning (Finn et al., 2013); and perceptual narrowing in infancy for cross-species voice perception (Friendly et al., 2013). This broader conceptualization of sensitive period effects is also illustrated by work examining the interaction of development and experience at different ages, including infancy (Bosseler et al., 2013; Weikum et al., 2013), early childhood (Bailey and Penhune, 2013; Putkinen et al., 2013) and even adulthood (Finn et al., 2013). Articles in this volume also examine sensitive period effects in the auditory and visual systems in relation to sensory loss or deprivation (Gordon et al., 2013; Voss, 2013). Sensitive period effects are being explored at a number of different levels of the nervous system, including work at the molecular and cellular levels. One study examines how the interaction of normal brain development and the timing of gene expression may explain pathology in developmental disorders (Kroon et al., 2013) and another paper reviews work using a rat model to study how the timing of a perinatal insult affects later auditory processing (Fitch et al., 2013). Finally, because complex experience impacts brain systems involved in multiple processes, a number of papers examine transfer across domains, especially the effect of musical training on language processing (Martínez-Montes et al., 2013; Putkinen et al., 2013; White et al., 2013).

Taken together, the articles selected for this Special Topic are outstanding examples of the range of questions and approaches that characterize the new approach to studying sensitive period effects today. We hope that they will provide both an empirical background and theoretical basis for future work.

Based on the research presented here, we see a number of broad questions and challenges to be addressed by future research into sensitive periods. These include: (1) generating new information about the neurobiological and experiential mediators of structural and functional brain changes; (2) proposing models of brain development that better predict when sensitive periods should occur and what functions would be implicated; (3) investigation of the interaction between experience during a sensitive period and pre-existing individual differences; (4) examining the relationship between experience during a sensitive period and on-going experience and (5) determining the mechanisms by which sensitive period-like plasticity could be re-activated in the adult brain for the remediation of perceptual or cognitive impairments.

## Conflict of interest statement

The authors declare that the research was conducted in the absence of any commercial or financial relationships that could be construed as a potential conflict of interest.
